# Investigating time-based expectancy beyond binary timing scenarios: evidence from a paradigm employing three predictive pre-target intervals

**DOI:** 10.1007/s00426-021-01606-2

**Published:** 2021-10-27

**Authors:** Stefanie Aufschnaiter, Fang Zhao, Robert Gaschler, Andrea Kiesel, Roland Thomaschke

**Affiliations:** 1 Cognition, Action and Sustainability Unit, Department of Psychology, Albert-Ludwigs-Universitaet Freiburg, Engelbergerstrasse 41, 79085 Freiburg, Germany; 2 Research Cluster D2L2, University of Hagen, Universitaetsstrasse 27, 58097 Hagen, Germany; 3 Department of Psychology, University of Hagen, Universitaetsstrasse 33, 58084 Hagen, Germany

## Abstract

When the duration of a pre-target interval probabilistically predicts the identity of the target, participants typically form time-based expectancies: they respond faster to frequent interval-target combinations than to infrequent ones. Yet, previous research investigating the cognitive time-processing mechanisms underlying time-based expectancy assessed time-based expectancy always in situations with a binary set of intervals (i.e. short vs. long). Here we aim to test whether time-based expectancy transfers to more complex settings with three different predictive time intervals (short, medium, long) in which each predicts one of three different target stimuli with 80% probability. In three experiments we varied how the medium interval was computed (arithmetic mean, geometric mean, or in between both). Our results showed that participants were able to learn the time-event contingencies for the short and the long as well as for the medium interval, and were, thus able to flexibly redirect their target expectancy two times during the course of a trial. The evidence concerning the impact of the manipulation of the medium intervals’ absolute duration on time-based expectancy was, however, mixed, as time-based expectancy for the medium interval could only be observed in one of three reported experiments. In sum, the findings of the present study suggest a previously unknown cognitive flexibility underlying time-based expectancy and offer important theoretical implications, challenging future research on the timing mechanisms involved in time-based expectancy.

## Investigating time-based expectancy beyond binary timing scenarios: evidence from a paradigm employing three predictive pre-target intervals

It is widely known that human action especially benefits from prediction when being confronted with situations, which require multitasking (Broeker et al., 2020; Gaschler et al., 2018). Apart from various other sources of predictability in the context of multitasking (for a comprehensive overview, see Broeker et al., 2017), the human cognitive system can benefit from the temporal distribution of tasks. Previous research revealed that individuals are able to form time-based expectancies, when one specific time interval co-occurs frequently with a specific target stimulus (Thomaschke et al., 2015; Volberg & Thomaschke, 2017). This is evidenced by shorter response times for frequent than for infrequent combinations of interval and target stimulus. Thus, time-based expectancy implies the processing of the length of the time interval preceding a target stimulus as a source of expectancy concerning the identity of the following target stimulus.^1^

In controlled experimental settings, time-based expectancy is typically investigated by applying the time-event correlation paradigm, initially introduced by Wagener and Hoffmann (2010). The time-event-correlation paradigm represents an adaptation of the variable foreperiod (FP)^2^ paradigm. Compared to established variable FP paradigms, which do not employ any contingencies between FP and target stimuli, in the time-event correlation paradigm one of two possible target stimuli is frequently combined with the short FP, whereas, the other target stimulus is frequently combined with the long FP preceding the onset of the target stimulus. Please note that each of the two different target stimuli and each of the two different FPs appear equally often in the time-event correlation paradigm. Typically, participants respond faster in trials with frequent combinations of FP and target compared to trials with infrequent combinations of FP and target, which is commonly referred to as a time-based expectancy effect^3^ (Thomaschke et al., 2011b).^4^

Although representing a ubiquitous phenomenon in our everyday lives, time-based expectancy has only been investigated since about ten years and therefore studies in this context constitute a relatively young line of cognitive research on human timing abilities. However, the few empirical studies hitherto investigating time-based expectancy have in common that they were unanimously able to prove time-based expectancy as a robust cognitive phenomenon by all means: time-based expectancy could be shown for affective dimensions (Thomaschke et al., 2018), in the context of human–computer interaction (Thomaschke & Haering, 2014), for conflict likelihood in an Eriksen flanker task (Wendt & Kiesel, 2011), as well as in the context of memory (Thavabalasingam et al., 2016; van de Ven et al., 2017) and language processing (Roberts & Francis, 2013; Roberts et al., 2011). For example, when posing a request in a conversation, the duration of the silent gap before the respondent answers, determines which type of answer one expects. When the inter-turn silence in such a situation passes a certain duration, expectancy for a confirmative answer changes to expectancy for a rejection (Roberts & Francis, 2013). Moreover, several studies revealed that time-based expectancy probably facilitates post-perceptual processing stages (Thomaschke & Dreisbach, 2013; Thomaschke et al., 2011a; Volberg & Thomaschke, 2017), and also perceptual processing stages (Thomaschke et al., 2016; however, see Thomaschke et al., 2018). Furthermore, Aufschnaiter et al. (2018a) were able to demonstrate that not only specific responses in a single task scenario, but also task types in a task switching scenario (for a review on task switching, see Kiesel et al., 2010) can be expected based on time.

Surprisingly, all previous studies dealing with time-based expectancy, be it in the context of single-task (see Thomaschke & Dreisbach, 2013; Thomaschke et al., 2011a, 2011b) or multitasking scenarios (see Aufschnaiter et al., 2018a; Jurczyk et al., 2020), employed only one short and one long predictive FP per experiment so far. Nonetheless, authors in the time-based expectancy literature frequently emphasize the practical importance of the phenomenon and its pervasiveness in everyday life (Aufschnaiter et al., 2018a). Thus, one might conjecture that time-based expectancy is restricted to two-time FPs. This suspicion is further corroborated by recent studies which have shown that the inner clock underlying time-based expectancy is a categorical, not a metric one (Aufschnaiter et al., 2018b; Thomaschke et al., 2015), which also suggests that time-based expectancy might be restricted to such binary timing scenarios. This would mean that the events being expected based on time are associated to a timing mechanism, which can only differentiate between a relatively short and a relatively long FP, but is insensitive to the absolute duration of an FP. Of course, the assumption that time-based expectancy is based on relative representations of time does not necessarily mean that is has to be conceived of as following a binary logic. However, it should be noted at this point that often aspects of our environments are perceived primarily in binary categories or sequences (see Oskarsson et al., 2009). Thus, in many contexts our cognition fails or at least encounters difficulties whenever a medium category has to be mentally represented (see Oskarsson et al., 2009).

Given these implications, it seems to remain an open question, if it is possible to exploit the predictive value of three different FPs in one timing scenario—although real-life timing scenarios might suggest that this is possible.^5^ Please note that this would mean that participants would have to learn three different associations between FP and stimulus (or task) instead of only two as it has been the case in previous studies on time-based expectancy.

## Aim of the present study

The present study investigates if humans are able to differentially associate three different events to three different predictive FPs. Each of the three FPs (short, medium and long) was predictive (80%) for one of three possible imperative stimuli, requiring a specific motor response.

Based on the clear-cut findings of previous studies (Aufschnaiter et al., 2018b; Thomaschke et al., 2015), we expected participants to learn the associations between the short FP and its corresponding stimulus, as well as for the long FP and its corresponding stimulus, and to build time-based expectancies for these two stimuli. However, concerning the medium FP, we did not have any directed hypotheses, because it was not clear beforehand, if time-based expectancy was restricted to binary timing scenarios (i.e. with only a short and a long FP) and if participants would be able at all to make use of the predictive value of three pre-target FPs of different duration.

It is important to note that time-based expectancy is an implicit phenomenon, which means that participants usually are not aware about the contingencies between FP and imperative stimulus. However, although the cognitive processing of the predictive pre-target FP happens outside of participants’ consciousness, participants have to discriminate between FPs of different absolute length to build up time-based expectancies for different FPs. To create optimal experimental conditions for time-based expectancy to occur with three FPs, we aimed at making the middle FP maximally distinct from both the short and the long FP. Yet, determining this difference is not possible without ambiguity, because literature from the area of absolute time judgements allows different conclusions regarding the “subjective” middle between two FPs: previous research has shown that the point, at which non-human animals are not able to discriminate clearly between the duration of a short and a long FP, referred to as the bisection point, is located at the geometric mean of the two referent durations (Church & Deluty, 1977). Interestingly, studies involving human subjects have found the bisection point to be located rather at the arithmetic, not at the geometric, mean between two durations (Droit-Volet & Wearden, 2001; Wearden, 1991; however, see Allan & Gibbon, 1991, and Provasi et al., 2011). Please note, however, that some previous studies were able to show an influence by the ratio of the short and the long FP as well as the duration range on human bisection performance (cf. Allan, 2002; Wearden & Ferrara, 1996). Thus, there is no clear-cut evidence on an optimal bisection point based on previous data so far (for a comprehensive meta-analysis on human performance on the temporal bisection task, see Kopec & Brody, 2010).

As previous research suggests that time-based expectancy does not rely on absolute timing mechanisms (see above), and as we supposed that cognition might encounter difficulties when a medium category has to be mentally represented (see Oskarsson et al., 2009), we devised three different experiments to avoid confining ourselves to a potentially suboptimal medium FP duration. The reported experiments only differed from each other regarding the length of the medium FP. Whereas, the short (200 ms) and the long (1800 ms) FPs were held constant in all three experiments, the medium FP constituted the arithmetic mean (1000 ms) in Experiment 1, whereas, it constituted the geometric mean (600 ms) in Experiment 2, and finally the average (800 ms) of the arithmetic and the geometric mean in Experiment 3.

## Experiment 1

To investigate whether time-based expectancy for stimulus–response events is possible with three predictive pre-target FPs, a time-event correlation paradigm with three different pre-target FPs (200 ms, 1000 ms, 1800 ms) and three different stimuli was employed in Experiment 1. The medium FP constituted the arithmetic mean of the short and the long FP. Each of the three possible FPs predicted one of the stimuli with 80% probability.

### Method

#### Participants

36 participants (26 females; mean age 26.28, SD 5.53, range 20–51 years; 33 right-handed) were tested in exchange for monetary compensation or course credit. Participants were students of the university of Freiburg or inhabitants of the city of Freiburg, who had normal or corrected-to-normal vision and were naïve concerning the hypotheses. Furthermore, only participants, who had not yet taken part in a comparable experiment about time-based expectancy before, were eligible for the present experiment. Participants were treated according to the ethical standards of the American Psychological Association and provided written informed consent prior to the experiment.

#### Apparatus and stimuli

Right-handed participants responded with the index finger, the middle finger and the ring finger of their right hand. Left-handed participants responded with the ring finger, the middle finger and the index finger of their left hand on the keys “g”, “z” and “j” on a QWERTZ keyboard. The keyboard was centrally aligned in front of the computer screen. Target stimuli were a square, a circle and a triangle (white line-shaped), presented against a black background at a viewing distance of 50 cm. The size of the stimuli was approximately 8 × 8 mm. The fixation cross was the plus symbol (Arial typeface, approximately 5 × 5 mm). All stimuli were presented centrally on the screen (refresh rate: 144 Hz).

#### Procedure

Each trial started with a blank screen for 300 ms (inter-trial FP), which was followed by the presentation of a fixation cross for a variable FP of either 200 ms, 1000 ms or 1800 ms. After this pre-target FP the target stimulus was presented. There was no time limit for responding to target stimuli, and stimuli remained on screen until button press. The order of stimuli was randomized, and each stimulus occurred with equal probability. Yet, each stimulus was preceded in 80% of its occurrence by a specific pre-target FP. Depending on the type of the stimulus, participants had to press a certain key. The mapping of keys to stimuli was counterbalanced across participants. Participants were instructed to respond as fast and as correctly as possible. After the detection of an error, the word *Fehler!* (German for “Error!”) was displayed in red on a black screen for 1500 ms. After correct responses, no explicit feedback was given.

The experiment took 45 min, and was composed of six blocks: one practice block and five experimental blocks. Each block comprised 120 trials. The only difference between the practice block and the remaining blocks was that after the detection of an error, the instruction was once again presented in silver font color on a black screen for 8000 ms in the practice block, before the next trial started with the presentation of the inter-trial FP. Between blocks, participants could take a break, which they could terminate individually by pressing the spacebar. In all blocks, the duration of the pre-target FP predicted the upcoming stimulus in the current trial with 80% probability, while in the remaining 20% of the trials the two other stimuli occurred in 10% each. Thus, each of the three stimuli occurred frequently after one of the three FPs. All FPs as well as all stimuli appeared with the same overall frequencies, and the mapping of FPs to stimuli was counterbalanced across participants. Participants were not informed that the pre-target FPs had different lengths, or that these FP lengths were correlated with the above-mentioned stimuli.

Data from the practice block, from the first three trials of each of the remaining blocks, as well as trials following an error trial were excluded from analyses. In addition, we excluded trials with RTs < 100 ms from analyses.

Furthermore, we removed trials in which RTs deviated more than three standard deviations from the corresponding cell mean, computed separately for each participant and experimental condition before RT analyses (Bush et al., 1993). In addition, trials with errors were removed from RT analyses.

Bayesian analyses with default prior scales were conducted whenever appropriate using JASP (0.8.1.1). The Bayesian approach is a model selection procedure that indicates the likelihood ratio of two or more hypotheses based on the given data. Bayesian analysis provides the possibility of evaluating evidence in favor of the (null-) hypothesis. In this regard, a Bayes factor (BF) in the range of 3–10 indicates moderate evidence in favor of the null-hypothesis (H0), respectively, the alternative hypothesis (H1) (Lee & Wagenmakers, 2013).

### Results

Overall, 24.8% of the trials were excluded from the RT analysis for Experiment 1 due to data preprocessing.

First, we calculated a repeated measures ANOVA with the within-subjects factors “FP duration in current trial” (200 ms, 1000 ms, 1800 ms; from here on, we will refer to this factor as “current FP”) and the factor “frequent FP for displayed stimulus in current trial” (200 ms, 1000 ms, 1800 ms; from here on, we will refer to this factor as “frequent FP”). The Greenhouse–Geisser adjustment was used to correct for violations of sphericity whenever necessary (an asterisk will mark the corresponding adjustments).

**Fig. 1:**
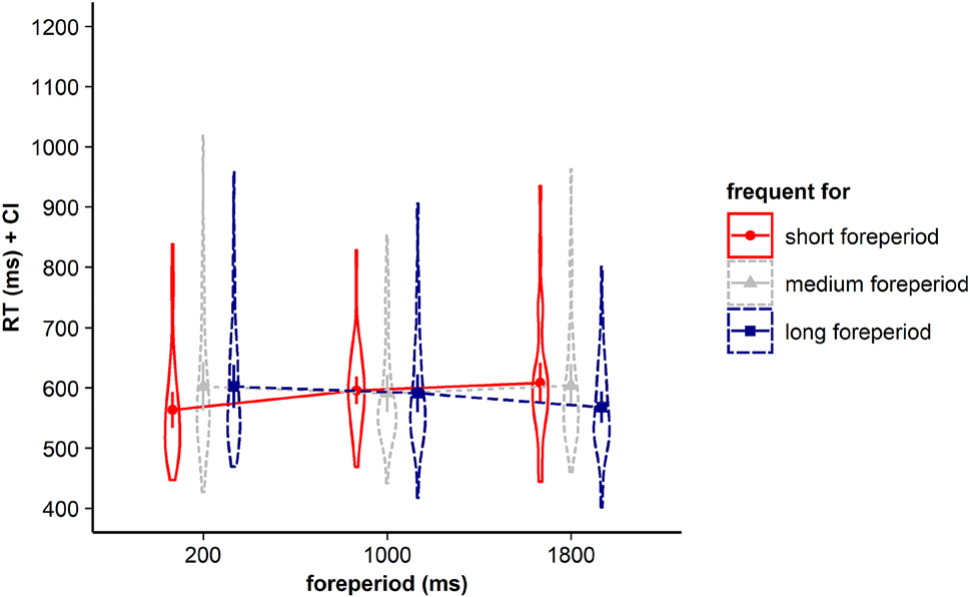
Violin plots represent data distribution of reaction times (RTs) of all participants in Experiment 1, depending on current foreperiod (FP) duration (200 ms, 1000 ms, 1800 ms) and frequent FP for displayed stimulus in the current trial (frequent for the short, the medium, or the long FP). The error bars of the corresponding means represent the respective confidence intervals

Results revealed that neither the main effect for current FP, *F* < 1, BF in favor of H0 = 24.917, nor the main effect for frequent FP, *F* < 1, BF in favor of H0 = 9.235, gained significance. However, the interaction between the two factors gained significance, *F*(4, 140) = 11.82, *p* < 0.001, *η*_*p*_^2^ = 0.252*, BF in favor of H1 = 36.230. Corresponding RTs are displayed in Fig. 1 and Table 1.

**Table 1:** Mean reaction times and SD for each factor combination in Experiment 1

Frequent FP for displayed stimulus in current trial						
FP of current trial	200		1000		1800	
	*M* (ms)	SD	*M* (ms)	SD	*M* (ms)	SD
200	563	89	602	117	602	105
1000	595	68	591	90	592	93
1800	609	99	604	105	568	77

**Fig. 2:**
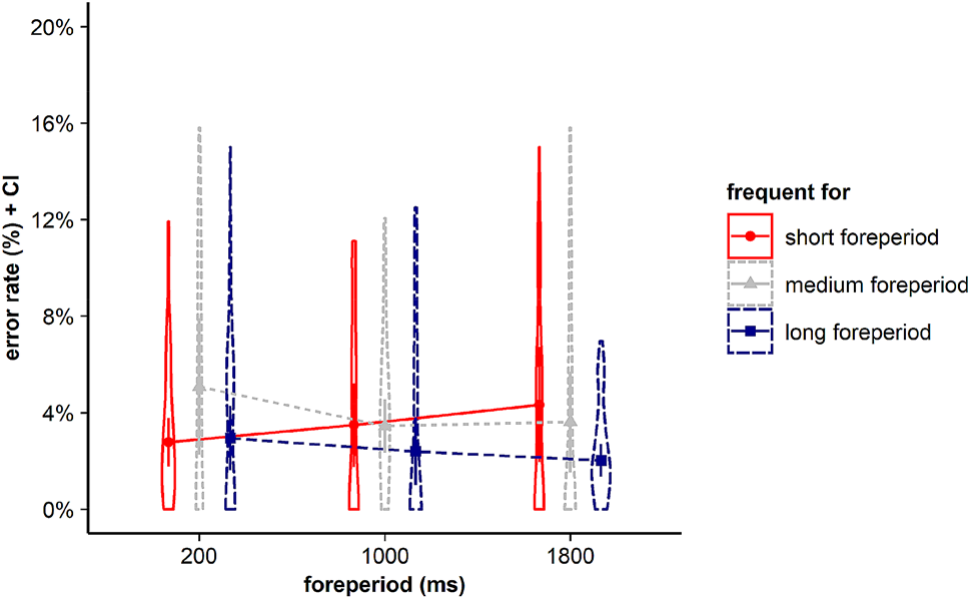
Violin plots represent data distribution of error rates of all participants in Experiment 1, depending on current foreperiod (FP) duration (200 ms, 1000 ms, 1800 ms) and frequent FP for displayed stimulus in the current trial (frequent for the short, the medium, or the long FP). The error bars of the corresponding means represent the respective confidence intervals

For error rates, results revealed no significant main effects for current FP, *F* < 1, BF in favor of H0 = 23.346, and frequent FP, *F*(2, 70) = 2.962, *p* = 0.058, *η*_*p*_^2^ = 0.078, BF in favor of H0 = 0.736. Furthermore, the interaction between the within-subjects factors current FP and frequent FP did not gain significance, *F*(4, 140) = 1.815, *p* = 0.153, *η*_*p*_^2^ = 0.049*, BF in favor of H0 = 5.696 (see Fig. 2).

**Fig. 3:**
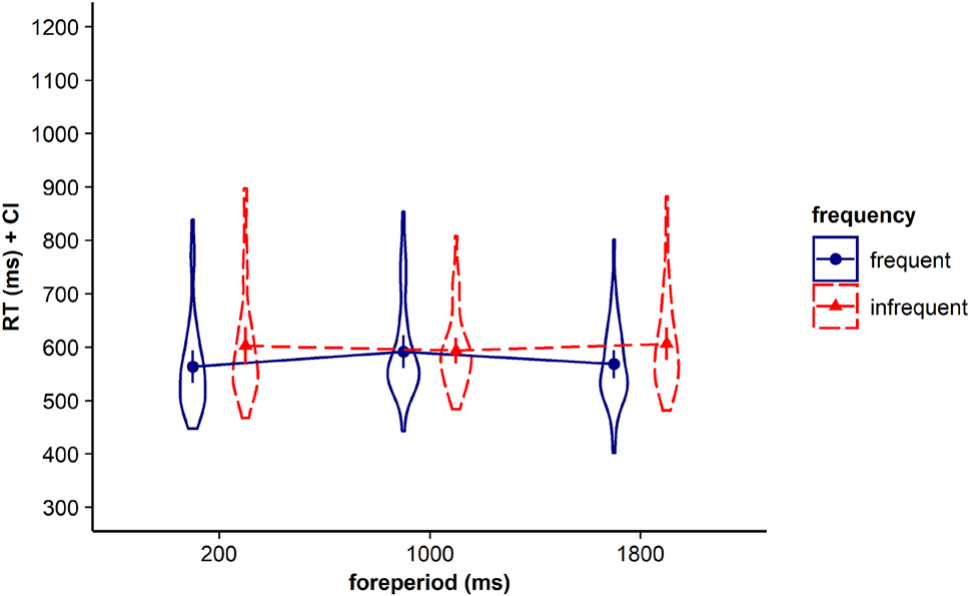
Violin plots represent data distribution of reaction times (RTs) for all participants in Experiment 1, depending on foreperiod (FP) duration (200 ms, 1000 ms, 1800 ms) and frequency of combination of FP and target stimulus (frequent, infrequent). The error bars of the corresponding means represent the respective confidence intervals

Visual inspection of Fig. 1 and Table 1 showed that for all three FP durations, including the medium FP, participants showed the fastest RTs for stimuli that had been frequently paired with the respective FP before. To determine whether participants indeed showed a significant time-based expectancy effect for each of the three FPs we conducted follow-up *t*-tests. Please note that to test if participants were able to build up time-based expectancy with all three employed FP durations, we aggregated data for infrequent trials. For example, for the short FP, this means that we tested performance in trials with frequent combinations of FP and stimulus (i.e. trials with short FP and the frequently associated stimulus) against infrequent combinations of FP and stimulus (i.e. trials with the short FP and the stimuli being frequently associated with the medium or the long FP). For the medium FP of 1000 ms, we did not find a significant difference between RTs in trials with frequent FP–stimulus combinations and RTs in trials with infrequent FP–stimulus combinations, *t*(35) = 0.147, *p* = 0.884, BF in favor of H0 = 5.529 (see Fig. 3). Consistent with the hypothesis-driven analysis, we conducted exploratory *t*-tests for the short and the long FP. Our results revealed that for the short FP, mean RTs in trials with frequent combinations of FP and stimulus were significantly faster than mean RTs in trials with infrequent combinations of FP and stimulus, *t*(35) = 3.674, *p* < 0.001, *d*_*z*_ = 0.612, BF in favor of H1 = 38.77. For the long FP of 1800 ms, mean RTs in trials with frequent combinations of FP and stimulus also were significantly faster than mean RTs in trials with infrequent combinations of FP and stimulus, *t*(35) = 3.454, *p* = 0.001, *d*_*z*_ = 0.576, BF in favor of H1 = 22.48.

### Discussion

Results of Experiment 1 replicated previous findings on time-based expectancy for two FPs and extended these findings by showing that time-based expectancy seems also possible in a time-event correlation paradigm with three different predictive FPs. However, Experiment 1 also suggests that participants benefit less from time-based expectancy at the medium FP, as follow-up *t *tests showed that the time-based expectancy effect was only significant for the short and the long FPs. However, also at the medium FP, participants responded numerically fastest to the stimulus that was frequently paired with it.

Yet, finding that the frequent pairing of long FP and stimulus as well as short FP and stimulus leads to RT benefits in a setup with three FPs and three stimuli is important in and of itself. It helps to rule out an alternative interpretation of the frequency effect in previous studies with only two FPs. It would have been conceivable that performance benefits in these studies were based on only learning one stimulus-FP pairing: People would prepare for the response of the stimulus often paired with the short FP as long as this has not passed by and prepare to press the other key instead as soon as the short FP has passed.

The next step after showing that the time-based expectancy effect for short and long FPs is present even with three FPs and stimuli is to test whether learning for the middle FP might be conditional upon the specific timing. To investigate, whether participants are able to build time-based expectancies in a paradigm with three predictive pre-target FPs if the medium FP constitutes the geometric (and not the arithmetic mean, as it was the case in Experiment 1), we conducted Experiment 2.

## Experiment 2

As to date, studies on human timing performance have yielded ambiguous results concerning the subjective estimation of a bisection point, sometimes revealing that the subjective midpoint of two referent durations is not perceived to be at the arithmetic mean of these two durations, but rather appears to be perceived at the geometric mean (see Allan & Gibbon, 1991; Provasi et al., 2011), the absolute length of the medium FP was defined as the geometric mean of the short and the long FP in Experiment 2 (instead of the arithmetic mean, which was the case in Experiment 1). The geometric mean of two durations is defined as the square root of the product of the two referent durations. As the short and the long duration were held constant in Experiment 2 with regard to Experiment 1, the geometric mean of these two durations was identified at 600 ms (√(200 × 1800) = 600).

### Method

#### Participants

36 participants (23 females; mean age 24.03, SD 3.04, range 20–33 years; 31 right-handed) took part in the experiment. Participants were again students from the University of Freiburg or inhabitants of the city of Freiburg, who received course credits or monetary compensation for their participation. All participants were unique to the experiment and fulfilled the same criteria as in Experiment 1.

#### Apparatus, stimuli, and procedure

Apparatus, stimuli and procedure were the same as in Experiment 1, with the exception that instead of the arithmetic mean of the short and the long FP (see Experiment 1), the duration of the medium FP was defined as the geometric mean of the short and the long FP in Experiment 2.

### Results

Data preprocessing was the same as in Experiment 1. Overall, 25.1% of the trials were excluded from the RT analysis for Experiment 2. RT and PE analysis were also conducted as in Experiment 1.

**Fig. 4:**
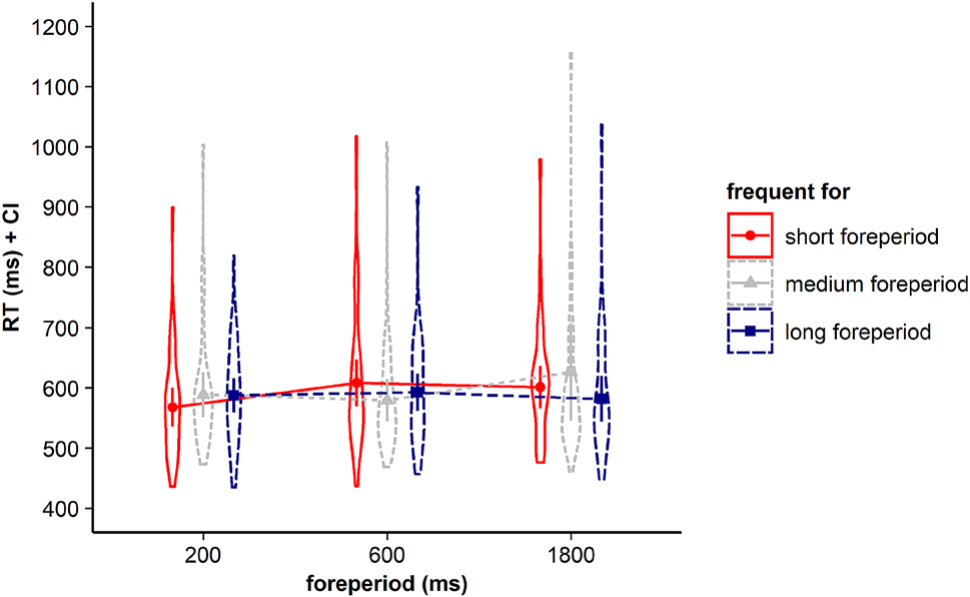
Violin plots represent data distribution of reaction times (RTs) of all participants in Experiment 2, depending on current foreperiod (FP) duration (200 ms, 600 ms, 1800 ms) and frequent FP for displayed stimulus in the current trial (frequent for the short, the medium, or the long FP). The error bars of the corresponding means represent the respective confidence intervals

Again, we first calculated a repeated measures ANOVA with the within-subjects factors current FP and frequent FP. Results revealed neither a main effect for current FP, *F*(2, 70) = 2.95, *p* = 0.079, *η*_*p*_^2^ = 0.078*, BF in favor of H0 = 2.528, nor a main effect for frequent FP, *F* < 1*, BF in favor of H0 = 13.286. However, the interaction between the two factors reached significance, *F*(4, 140) = 4.196, *p* = 0.031, *η*_*p*_^2^ = 0.107*, BF in favor of H1 = 1.933. Corresponding RTs are displayed in Fig. 4 and Table 2.

**Table 2:** Mean reaction times and SD for each factor combination in Experiment 2

Frequent FP for displayed stimulus in current trial						
FP of current trial	200		600		1800	
	*M* (ms)	SD	*M* (ms)	SD	*M* (ms)	SD
200	568	95	590	110	587	84
600	609	114	579	103	591	91
1800	603	103	629	239	581	112

**Fig. 5:**
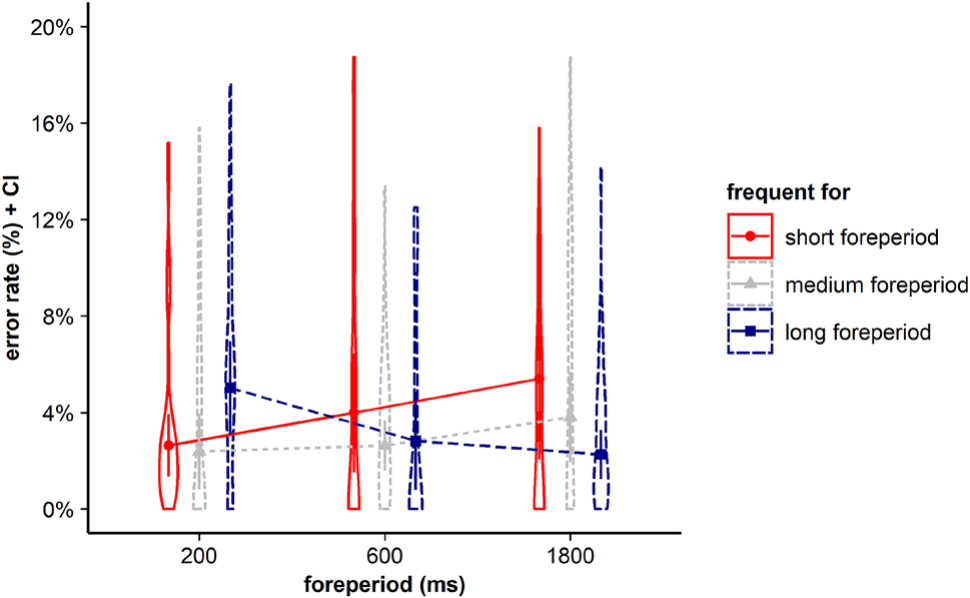
Violin plots represent data distribution of error rates of all participants in Experiment 2, depending on current foreperiod (FP) duration (200 ms, 600 ms, 1800 ms) and frequent FP for displayed stimulus in the current trial (frequent for the short, the medium, or the long FP). The error bars of the corresponding means represent the respective confidence intervals

For error rates, results revealed no significant main effects for the ANOVA with the within-subjects factors current FP, *F*(2, 70) = 1.389, *p* = 0.256, *η*_*p*_^2^ = 0.038, BF in favor of H0 = 12.396, and frequent FP, *F*(2, 70) = 1.822, *p* = 0.169, *η*_*p*_^2^ = 0.049, BF in favor of H0 = 3.609. However, the interaction between current FP and frequent FP gained significance, *F*(4, 140) = 5.553, *p* = 0.001, *η*_*p*_^2^ = 0.137*, BF in favor of H1 = 64.225 (see Fig. 5).

Visual inspection of Fig. 4 and Table 2 shows that for all three FP durations, including the medium FP, participants showed the fastest RTs for stimuli that had been frequently paired with the respective FP before. To determine whether participants indeed showed a time-based expectancy effect for each of the three FPs, we conducted follow-up *t* tests. Please note that again, we aggregated data for infrequent trials (see Experiment 1).

**Fig. 6:**
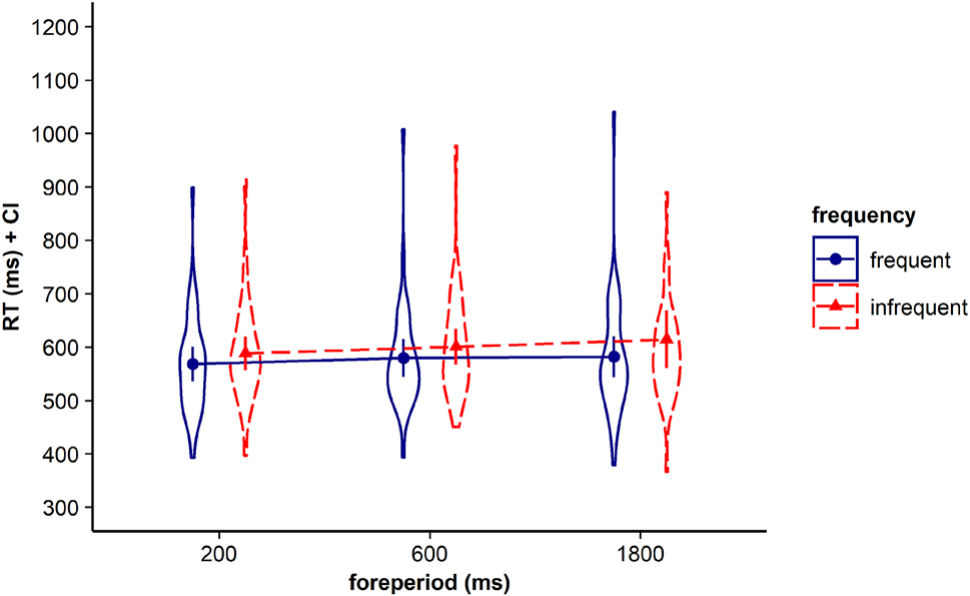
Violin plots represent data distribution of reaction times (RTs) for all participants in Experiment 2, depending on foreperiod (FP) duration (200 ms, 600 ms, 1800 ms) and frequency of combination of FP and target stimulus (frequent, infrequent). The error bars of the corresponding means represent the respective confidence intervals

Results of a follow-up *t* test showed that after the medium FP, participants responded faster in trials with frequent FP–stimulus combinations than in trials with infrequent FP–stimulus combinations, *t*(35) = 2.608, *p* = 0.013, *d*_*z*_ = 0.435, BF in favor of H1 = 3.322 (see Fig. 6). Consistent with the hypothesis-driven analysis, we again conducted exploratory analyses, which revealed that participants responded faster in trials with frequent FP–stimulus combinations than in trials with infrequent FP–stimulus combinations also after the short FP, *t*(35) = 3.317, *p* = 0.002, *d*_*z*_ = 0.553*,* BF in favor of H1 = 16.14, as well as after the long FP, *t*(35) = 2.091, *p* = 0.044, *d*_*z*_ = 0.349, BF in favor of H1 = 1.239.

**Fig. 7:**
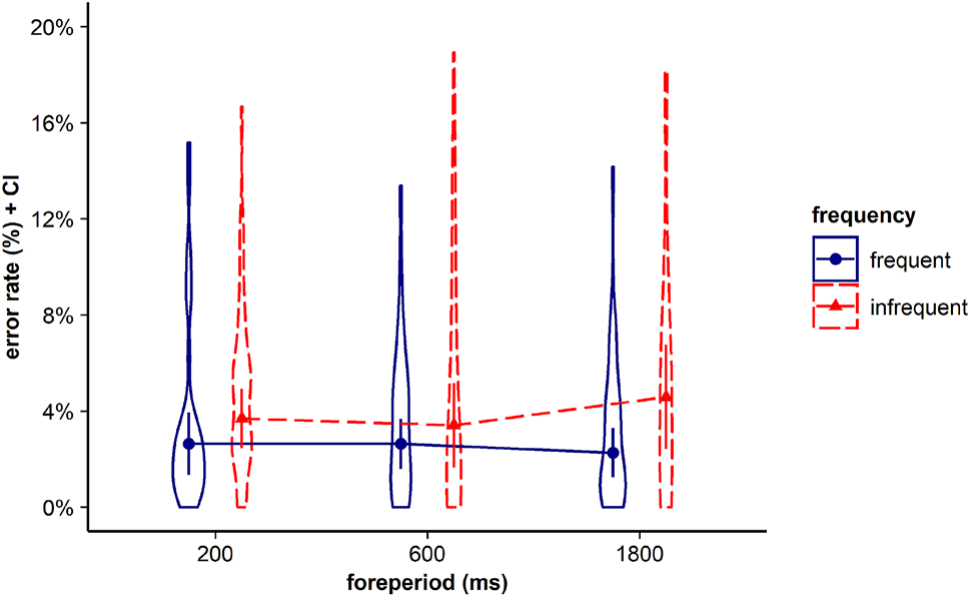
Violin plots represent data distribution of error rates for all participants in Experiment 2, depending on foreperiod (FP) duration (200 ms, 600 ms, 1800 ms) and frequency of combination of FP and target stimulus (frequent, infrequent). The error bars of the corresponding means represent the respective confidence intervals

With regard to error rates, the difference between mean error rates in trials with frequent combinations of FP and stimulus and mean error rates in trials with infrequent combinations of FP and stimulus did not gain significance for the medium FP, *t*(35) = 1.490, *p* = 0.145, BF in favor of H0 = 2.036. Exploratory analyses, however, revealed for the short FP, *t*(35) = 2.042, *p* = 0.049, *d*_*z*_ = 0.340, BF in favor of H1 = 1.138, as well as for the long FP, *t*(35) = 3.302, *p* = 0.002, *d*_*z*_ = 0.550, BF in favor of H1 = 15.59, that the difference between mean error rates in trials with frequent FP–stimulus combinations and mean error rates in trials with infrequent FP–stimulus combinations gained significance (see Fig. 7).

### Discussion

Experiment 2 investigated if humans are able to build time-based expectancies for three predictive FPs in one experimental paradigm, if the medium FP constitutes the geometric mean of the short and the long FP. Results revealed a significant time-based expectancy effect for the short and the long, as well as for the medium FP. This means that participants were able to make use of the predictive value of each of the three FPs, indicated by showing faster responses in trials with frequent FP–stimulus combination after the short, the medium and the long pre-target FP.

To our knowledge, this is the first experiment to show that humans are able to flexibly redirect their time-based expectancy two times in trials employing a long FP and we conjecture the following sequence of expectations: at the start of the trial, participants expect the stimulus which frequently occurs at the short FP. When no stimulus is presented after the short FP has passed, expectancy changes in the direction of the stimulus associated with the medium FP. When the medium FP has also passed without any stimulus being presented, expectancy changes a second time—in the direction of the stimulus associated with the long FP. This rapid switch of expectancy, being obviously possible two times during a single trial course, reflects a highly flexible cognitive adaptation process.

To investigate how precisely the duration of the medium FP has to be chosen as the geometric mean as a premise so that humans are able to exploit the predictive value of each of three pre-target FPs in one experimental paradigm, we conducted Experiment 3.

## Experiment 3

The results of Experiment 1 and 2 have shown that participants seem to be able to use a medium FP for the formation of time-based expectancy in a paradigm with three predictive pre-target FPs only if this medium FP constitutes the geometric mean of the short and the long duration (and not the arithmetic mean, see Experiment 1). The purpose of Experiment 3 was to investigate, how restricted time-based expectancy for three FPs is with regard to the geometric mean of two referent durations. For this purpose, the medium FP constituted the average of the arithmetic (1000 ms) and the geometric (600 ms) mean in Experiment 3 and was thus defined at 800 ms.^6^

### Method

#### Participants

36 participants (20 females; mean age 32.36, SD 11.20, range 16—53 years; 35 right-handed) took part in the experiment. Participants were students from the University of Hagen or inhabitants of the city of Hagen, who received course credits or monetary compensation for their participation. All participants were again unique to the experiment and fulfilled the same criteria as in Experiments 1 and 2. One participant had to be excluded from analyses due to high error rates (mean percentage of errors: 11.74%; mean percentage of errors for all participants: 2.27%). This resulted in a final sample of 35 participants.

#### Apparatus, stimuli, and procedure

Apparatus, stimuli and procedure were the same as in Experiments 1 and 2, with the exception that the medium FP constituted the average of the arithmetic (1000 ms) and the geometric (600 ms) mean in Experiment 3, and was thus defined at 800 ms.

### Results

Data preprocessing was the same as in Experiments 1 and 2. Overall, 23.2% of the trials were excluded from the RT analysis for Experiment 3. RT and PE analysis were also conducted as in Experiments 1 and 2.

**Fig. 8:**
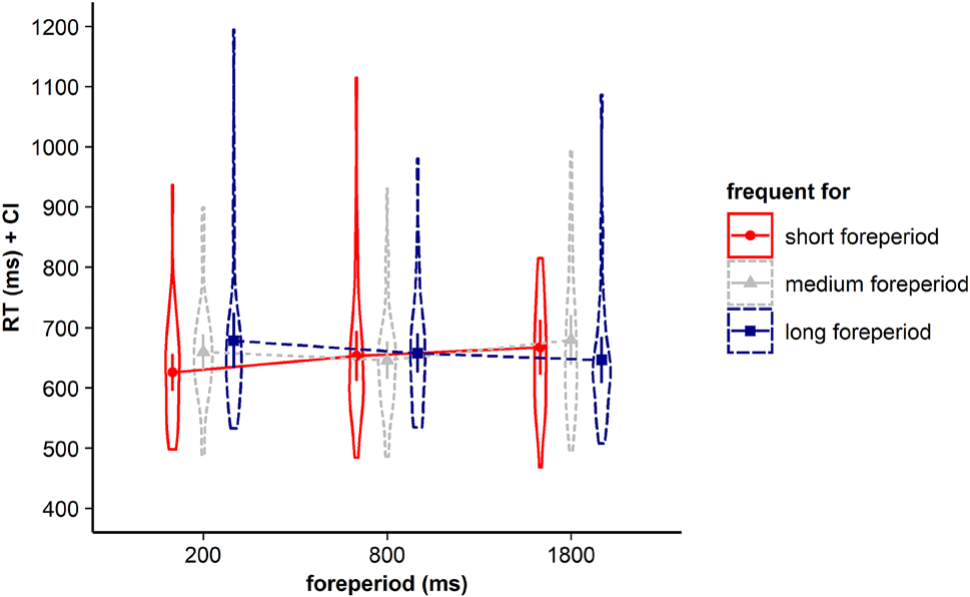
Violin plots represent data distribution of reaction times (RTs) of all participants in Experiment 3, depending on current foreperiod (FP) duration (200 ms, 800 ms, 1800 ms) and frequent FP for displayed stimulus in the current trial (frequent for the short, the medium, or the long FP). The error bars of the corresponding means represent the respective confidence intervals

The repeated measures ANOVA with the within-subjects factors current FP and the factor frequent FP revealed that neither the main effect for current FP, *F*(2, 68) = 1.462, *p* = 0.239, *η*_*p*_^2^ = 0.041, BF in favor of H0 = 10.167, nor the main effect for frequent FP, *F* < 1*, BF in favor of H0 = 10.322, gained significance. However, like in Experiments 1 and 2, the interaction between the two factors gained significance, *F*(4, 136) = 5.337, *p* = 0.002, *η*_*p*_^2^ = 0.136*, BF in favor of H1 = 5.542. Corresponding RTs are displayed in Fig. 8 and Table 3.

**Table 3:** Mean reaction times and SD for each factor combination in Experiment 3

Frequent FP for displayed stimulus in current trial						
FP of current trial	200		800		1800	
	*M* (ms)	SD	*M* (ms)	SD	*M* (ms)	SD
200	625	89	658	85	678	134
800	653	121	646	90	656	94
1800	668	133	680	120	646	112

**Fig. 9:**
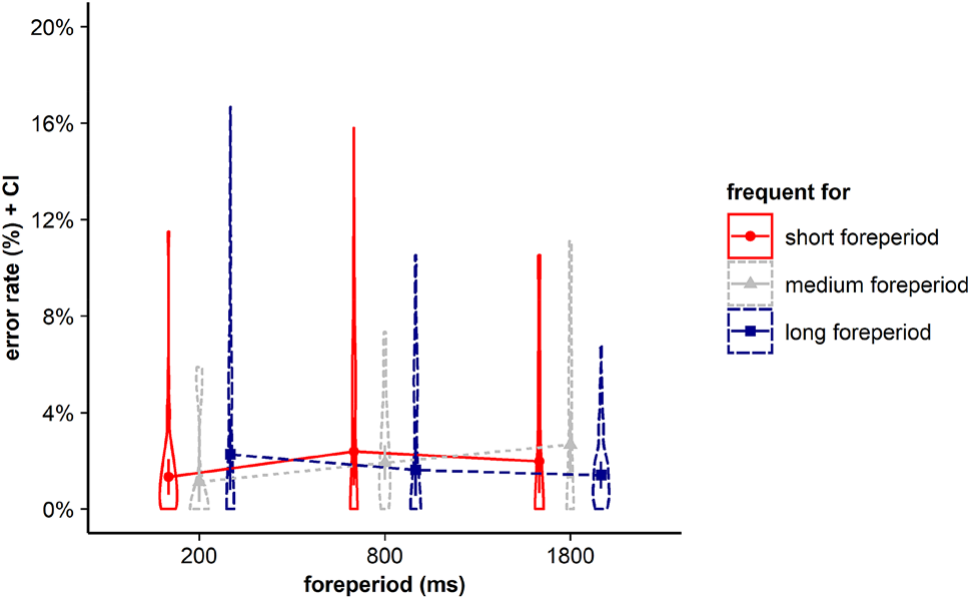
Violin plots represent data distribution of error rates of all participants in Experiment 3, depending on current foreperiod (FP) duration (200 ms, 800 ms, 1800 ms) and frequent FP for displayed stimulus in the current trial (frequent for the short, the medium, or the long FP). The error bars of the corresponding means represent the respective confidence intervals

For error rates, results revealed no significant main effects for current FP, *F*(2, 68) = 1.047, *p* = 0.357, *η*_*p*_^2^ = 0.030, BF in favor of H0 = 12.674, and frequent FP,* F* < 1*, BF in favor of H0 = 28.249. Furthermore, the interaction between the within-subjects factors current FP and frequent FP did not gain significance, *F*(4, 136) = 2.453, *p* = 0.061, *η*_*p*_^2^ = 0.067*, BF in favor of H0 = 1.211 (see Fig. 9).

Like in Experiments 1 and 2, visual inspection of Fig. 8 and Table 3 showed that for all three FP durations, including the medium FP, participants showed the fastest RTs for stimuli that had been frequently paired with the respective FP before. To determine whether participants indeed showed a time-based expectancy effect for each of the three FPs we conducted follow-up *t* tests. Again, we aggregated data for infrequent trials (see Experiment 1 and 2).

**Fig. 10:**
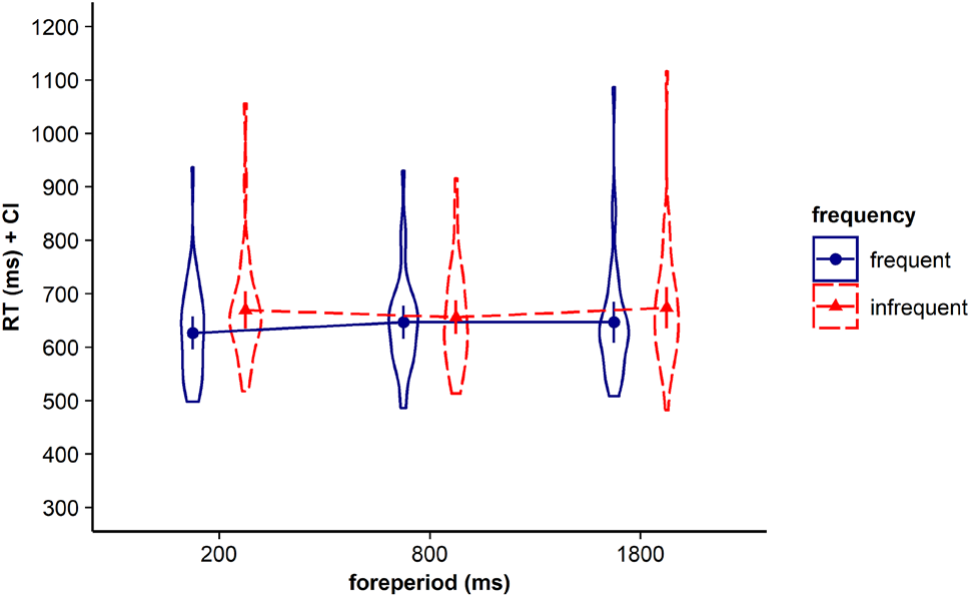
Violin plots represent data distribution of reaction times (RTs) for all participants in Experiment 3, depending on foreperiod (FP) duration (200 ms, 800 ms, 1800 ms) and frequency of combination of FP and target stimulus (frequent, infrequent). The error bars of the corresponding means represent the respective confidence intervals

Follow-up *t *tests revealed that for the medium FP of 800 ms, we did not find a significant difference between mean RTs in trials with frequent FP–stimulus combinations and trials with infrequent FP–stimulus combinations, *t*(34) = 0.890, *p* = 0.380, BF in favor of H0 = 3.824 (see Fig. 10). Consistent with the hypothesis-driven analysis, we conducted exploratory analyses, which, however, revealed faster RTs in trials with frequent combinations of FP and stimulus compared to trials with infrequent combinations of FP and stimulus for the short FP of 200 ms, *t*(34) = 4.009, *p* < 0.001, *d*_*z*_ = 0.678, BF in favor of H1 = 89.00, as well as for the long FP of 1800 ms, *t*(34) = 2.511, *p* = 0.017, *d*_*z*_ = 0.424, BF in favor of H1 = 2.735.

As results of the follow-up *t* tests of three reported experiments showed that we only found a significant difference between RTs in trials with frequent combinations of FP and stimulus, compared to trials with infrequent FP and stimulus, when the medium FP constituted the geometric mean of the short and the long FP (although in Exp. 1 and 3 a time-based expectancy effect was at least numerically observable for the medium FP), we conducted a cross-experiment analysis to investigate, if the time-based expectancy effect differs between experiments depending on the absolute duration of the respective pre-target FP. Results of the 3 × 3 × 3 ANOVA revealed a significant main effect for current FP, *F*(2, 208) = 3.557, *p* = 0.037, *η*_*p*_^2^ = 0.033*, BF in favor of H1 = 0.218. Neither the main effect for frequent FP, *F*(2, 208) = 1.168, *p* = 0.313, *η*_*p*_^2^ = 0.011, BF in favor of H0 = 11.079, nor the interaction between current FP and the between-subjects factor “experiment”, *F* < 1*, BF in favor of H0 = 87.537 gained significance. Also, the interaction between frequent FP and experiment was not significant, *F* < 1, BF in favor of H0 = 113.827. The main effect of the between-subjects factor experiment gained significance, *F*(2, 104) = 5.620, *p* = 0.005, *η*_*p*_^2^ = 0.098, BF in favor of H1 = 9.439. Most importantly, the interaction between current FP and frequent FP gained significance, *F*(4, 416) = 15.842, *p* < 0.001, *η*_*p*_^2^ = 0.132*, BF in favor of H1 = 220844.447, but, however, the interaction between current FP, frequent FP and experiment failed to reach significance, *F*(8, 416) = 1.010, *p* = 0.415, *η*_*p*_^2^ = 0.019*, BF in favor of H0 = 99.916.

For error rates, results only revealed a significant interaction between current FP and frequent FP, *F*(4, 416) = 6.492, *p* < 0.001, *η*_*p*_^2^ = 0.059*, BF in favor of H1 = 108.473, as well as a significant main effect for experiment *F*(2, 104) = 6.178, *p* = 0.003, *η*_*p*_^2^ = 0.106, BF in favor of H1 = 7.413.

### Discussion

Experiment 2 showed that humans are able to exploit the predictive value of three different pre-target FPs in one paradigm if the medium FP constitutes the geometric mean of the short and the long FP. To investigate, how precisely the medium FP has to be defined to be cognitively represented as an independent FP containing predictive information about the target stimulus, which is most likely to occur after this medium FP has passed, Experiment 3 was conducted: in Experiment 3, the medium FP was defined as the mean (800 ms) of the arithmetic mean (1000 ms; see Experiment 1) and the geometric mean (600 ms; see Experiment 2) of the short (200 ms) and the long (1800 ms) FP.

Results revealed a significant time-based expectancy effect for the short and the long FP, which means that participants responded significantly faster in trials with frequent FP–stimulus combinations, compared to trials with infrequent FP–stimulus combinations in those trials. Participants even showed (at least numerically) faster RTs in trials with the medium FP for the stimulus which was frequently paired with the medium FP, compared to when stimuli appeared, which were frequently paired with the short or the long FP.

A cross-experiment analysis over all three experiments revealed a significant interaction between current FP and FP frequently associated with the displayed stimulus in the current trial. This means that participants showed faster RTs for the stimulus which was frequently paired with the FP of the current trial. Importantly, this was—at least numerically—the case for every FP in each of the three reported experiments. Interestingly, this interaction between current FP and FP frequently associated with the displayed stimulus in the current trial did not differ between experiments, which was also strongly supported by Bayesian analysis.

## General discussion

The present study investigated, if time-based expectancy can be built for three predictive pre-target FPs of different absolute length in the time-event correlation paradigm. We reported three experiments, in which each of three pre-target FPs of different absolute length predicted one of three possible stimuli with 80% probability. As we aimed at making the middle FPs maximally distinct from both the short and the long FP to create optimal experimental conditions for time-based expectancy to occur with three predictive FPs, we based our preliminary considerations regarding the absolute duration of the medium FP on results of research on human performance in temporal bisection tasks. For this reason, the medium FP was chosen at the arithmetic mean in Experiment 1, whereas, it was chosen at the geometric mean in Experiment 2 and at the average of the arithmetic and the geometric mean in Experiment 3.

In all three reported experiments, we observed significant interactions between the FP duration in the current trial and the FP being frequently paired with the stimulus displayed in the current trial. Importantly, the data pattern for these interactions revealed that for every FP in each of the three experiments, participants showed faster RTs when FP duration of the current trial and FP duration being frequently paired with the stimulus presented in the current trial, matched. This means that participants in all three experiments responded faster, whenever the upcoming stimulus type could be predicted based on the pre-target FP than when a stimulus appeared, which had been frequently paired with one of the two other FPs before.

It should also be noted that the cross-experiment ANOVA revealed that participants showed fastest RTs in trials with the short FP and slowest RTs in trials with the long FP (although the pairwise analyses did not show any significant differences between the three FP durations). This finding is at odds with some previous studies on variable FPs (e.g., Los, 2010; Steinborn et al., 2008) showing opposite patterns. Possible explanations for this unexpected finding might draw on stimulus-induced shifts of phasic alertness (see also Aufschnaiter et al., 2018a; Meiran et al., 2000), or effects of time uncertainty (Klemmer, 1956; Näätänen, 1972). For example, Meiran et al. (2000) investigated effects of unspecific preparation by employing neutral warning signals before presenting the task cue. Although getting only weak effects, Meiran et al. (2000) assumed stimulus-induced shifts of phasic alertness as being involved in cognitive preparation processes. In the present study, the fixation cross before stimulus onset can be classified as a neutral signal, warning the participant that the target stimulus will be presented soon after (please note that of course, the duration of the fixation cross is informative about the following stimulus identity). Perhaps, phasic alertness, induced by the mere presentation of the fixation cross, might have contributed to overall shorter reaction times after the short FP (without interacting with effects of time-based expectancy), but might have decayed at the long FP. However, the observation of overall faster RTs after the short than after long FP could also be due to effects of time uncertainty, meaning that participants’ uncertainty regarding time estimation increases with increasing FP duration (cf. Klemmer, 1956; Näätänen, 1972).

We observed significant time-based expectancy effects in all three experiments for the short and the long FPs, and were thus able to replicate the findings of previous studies, which investigated time-based expectancy in the context of binary timing scenarios (Aufschnaiter et al., 2018b; Thomaschke et al., 2015). However, the result pattern is more mixed for the medium FPs: while we observed a significant time-based expectancy effect in Exp. 2, this effect did not reach significance in Exp. 1 and 3. Yet, a cross-experimental analysis revealed no difference of time-based expectancy for all three employed pre-target FPs between all three reported experiments. Thus, it seems that under certain circumstances, humans seem to be able to associate three different events with three predictive FPs of different absolute length in one experimental paradigm. However, given the fact that a significant time-based expectancy effect was only observable if the medium FP constituted the geometric mean, but the cross-experiment analysis, however, showed no significant difference between time-based expectancy for all employed pre-target FPs (short, medium and long) between experiments, no final conclusions can be drawn based on the present results. Rather, our results give a first indication that the absolute duration of the medium FP may have an influence regarding the degree of suitability of a medium FP to provide predictive information about upcoming task requirements. In this context, it would be a fruitful endeavor for future studies to investigate, how precisely the absolute length of the medium FP has to be chosen so that the predictive value of the medium FP can be exploited and be used for anticipatory redirection of cognitive control towards the stimulus, which is most likely to occur after the medium FP.

However, at this point, it should be noted that it might be also conceivable that participants encounter a high cognitive demand when being asked to track three stimuli and three different FPs. To our knowledge, no study has ever investigated the influence of cognitive load on time-based expectancy. Presuming that cognitive load was responsible for our results being rather mixed for the medium FP, it might be the case, that cognitive load could be diminished by extending the temporal distance between the absolute durations of the employed predictive FPs. Future studies might investigate, whether it is the duration of the medium FP in dependence on the durations of the short and the long FP, which has an impact on performance in time-based predictability given three different FPs. Or rather if it is the temporal distance between the three FPs, which might be connected to cognitive load, thus hampering the formation of time-based expectancy for each of three predictive FPs.

Importantly, please note, that the design of the present study does not allow to infer any assumptions concerning human timing performance on the bisection task, as we did not let participants explicitly judge time FPs, but rather determined the duration of the medium FP in all three reported experiments in advance to create optimal premises for the association of the three different pre-target FPs with the different target stimuli in each experiment. Furthermore, based on our findings, we cannot infer any assumptions about the timing mechanisms being involved in time-based expectancy. However, should future studies reveal clear-cut connections between the absolute duration of a predictive medium FP and its suitability to provide predictive information about upcoming task requirements, it might be a fruitful endeavor to conduct studies on human timing performance on a temporal bisection task and to correlate these findings on an individual level with participants’ performance in a time-event correlation paradigm, employing three predictive pre-target FPs. By doing so, it would be interesting to determine, if performance in an explicit temporal estimation task (i.e. the determination of the individual subjective bisection point) is linked to individual performance regarding the cognitive processing and exploitation of a predictive medium FP. It might be interesting in this context that Jozefowiez et al. (2018) recently posed the question if the representation of time, linearly vs. logarithmically,^7^ is rather dependent on the current experimental condition—the authors even assume in their article that there may exist individual differences regarding the type of time representation. However, please note that time-based expectancy is an implicit phenomenon, which means that participants are usually unaware of the predictive value of the pre-target FPs and seem to be, nevertheless, able to adapt their response behavior to the time-event contingencies which can be exploited by the cognitive system due to an associative learning mechanism (see Thomaschke & Dreisbach, 2015).

Most importantly, the finding of the present study that participants seemed to be able to make use of the predictive value of three pre-target FPs of different absolute length in one experimental paradigm constitutes a considerable extension of previous findings regarding the flexibility of time-based expectancy: in a recent study, Aufschnaiter et al. (2020) were able to show that time-based expectancy for tasks in a task-switching paradigm was even observable in an experiment, where both predictive FPs stemmed from the sub-second range (10 ms and 500 ms). In this context, the authors concluded that the updating of time-based expectancy seems to reflect a very fast and dynamic process, as expectancy switches rather fast towards the task associated with the long FP of 500 ms if no stimulus has been presented 10 ms after the onset of the warning signal. The present study even extends scientific knowledge regarding the cognitive flexibility underlying time-based expectancy: besides the finding of Aufschnaiter et al. (2020), who showed that participants are able to update their expectancy rather fast in a narrow temporal window during the course of a pre-target FP, the present study demonstrated that they seem to be also capable of redirecting their time-based expectancy two times instead of one time (as it was the case in previous studies on time-based expectancy, see Thomaschke & Dreisbach, 2015) during the time course of a trial employing a long FP: in Experiment 2 of the present study, participants expected Target 1, associated with the short FP. When the short FP had passed without any presentation of a target stimulus, expectancy changed towards the target associated with the medium FP. If the medium FP also had passed without any presentation of a target, expectancy changed (a second time) towards the stimulus associated with the long FP. This highly flexible redirection of time-based expectancy during the temporal course of a single trial points towards highly adaptable and therefore flexible cognitive mechanisms being involved in specific preparation processes, which underly time-based expectancy. It should be noted at this point that the fact that participants were able to switch their expectancy two times during trials employing a long FP, is quite remarkable, as previous studies could show that humans usually tend to avoid additional cognitive effort (Kool et al., 2010).

To conclude, the findings of the present study suggest that under certain circumstances, humans are able to exploit the predictive value of each of three pre-target FPs of different absolute duration regarding the upcoming imperative stimulus in one experimental paradigm. Our findings open up entirely new perspectives concerning cognitive flexibility by indicating that time-based expectancy does not seem to be restricted to binary timing scenarios (as recent studies investigating the type of time representation underlying time-based expectancy suggested), and therefore, offer practical implications regarding the applicability of time-based expectancy in real-life timing scenarios beyond laboratory settings. Furthermore, the present results challenge future research on the individual and contextual influences on human timing behavior underlying time-based expectancy. However, given that the time-based expectancy effect for the medium interval was only observed in one of the three reported experiments, future studies are required to investigate to what degree the absolute duration of the medium FP determines whether a medium FP is suitable to provide predictive information about upcoming task requirements.

## Footnotes

1 Please note that in contrast to time-based expectancy, which is the focus of the present study and which means anticipating what will happen and when it will happen, time expectancy means predicting when something will happen without exactly knowing what will happen at that point in time (Nobre & Coull, 2010; for a recent review on different paradigms investigating time expectancy, see Capizzi & Correa, 2018). Time expectancy is usually investigated by employing the variable foreperiod (FP) paradigm, in which the time span, called the FP, between the onset of the warning signal and the presentation of the target stimulus differs between trials within one experimental block (Los et al., 2001; Schröter et al., 2015; Steinborn et al., 2009). As time expectancy and time-based expectancy usually do not interact, time expectancy will not be further discussed in the present study (see Aufschnaiter et al., 2018a; Aufschnaiter et al., 2021; Kunchulia & Thomaschke, 2016).

2 For the sake of consistency, we will use the term “foreperiod” instead of “time interval” from here on throughout the text.

3 Please note that in variable FP paradigms, usually the so-called variable-FP effect (Niemi & Näätänen, 1981) can be observed, meaning that reaction times (RTs) decrease with increasing FPs. However, although FPs of different absolute lengths are employed in the time-event-correlation paradigm, the variable-FP effect is not always observable. There were studies on time-based expectancy, which found the variable-FP effect (see, for example, Thomaschke et al., 2011a) while others did not (Aufschnaiter et al., 2018a).

4 In the context of time-based expectancy, we use the term “expectancy” in a very broad sense, strongly overlapping with the terms “prediction” and “preparation”. This is important to note, because our usage substantially deviates from the meaning of “expectancy” in everyday language and in other areas of psychology. With “expectancy for A” we mean a modulation of the cognitive system to process event A better than the non-modulated system would. It is important to note that we use “expectancy” for generically referring to any modulation. This generic use is common in the current timing literature. In earlier times, expectancy was rather used to refer specifically to modulations of the perceptual system only, while preparation referred to modulations of the motor system, and prediction referred to conscious, explicitly represented future knowledge (see also Thomaschke et al., 2016). Thus, we use the term “expectancy” in a broad sense, which encompasses all kinds of anticipative psychological phenomena. However, this under-specification is indeed on purpose, as we assume our findings to generalize by default to all domains of anticipative processing, as long as the predictability (i.e. events are predictable when they are redundantly distributed in a specific context; that is, their appearance is not completely random in that context) in those domains is based on time.

5 It should be noted, however, that apart from research on time-based expectancy, several studies on human timing abilities do exist, both from the area of subjective duration judgements and time expectancy, in which more than two FPs were employed in one experiment (cf. Cravo et al., 2011; Grondin & Rammsayer, 2003; Langner et al., 2018).

6 Please note that choice of the average of the geometric and the arithmetic mean of the short (200 ms) and the long FP (1800 ms) in Exp. 3 did not follow theoretical assumptions. Rather, it was chosen in an arbitrary manner, as we wanted to investigate, if time-based expectancy can be built for a medium FP, which was longer than the geometric mean (see Exp. 2), but shorter than the arithmetic mean (see Exp. 1).

7 For a comprehensive overview regarding the connection between representations of time, linearly vs. logarithmically, and content of learning, cognitive models vs. associative models, see Jozefowiez & Machado (2013).
